# A Study of the Clinico-Etiological Profile of Splenomegaly Among Children in a Tertiary Care Hospital

**DOI:** 10.7759/cureus.98299

**Published:** 2025-12-02

**Authors:** Kuppireddy Lakshmisindhu, Modalavalasa Sravanadurga, Noolu Ramalingeswara, Lanka Gowthami

**Affiliations:** 1 Pediatrics and Child Health, Great Eastern Medical School and Hospital, Srikakulam, IND

**Keywords:** conventional classification of splenomegaly, hacketts classification, hematological, infections, splenomegaly

## Abstract

Background

Splenomegaly, or enlargement of the spleen, is a clinical sign rather than a standalone diagnosis, often indicating underlying systemic disease. In children, it may present due to a broad spectrum of conditions ranging from infections to hematologic, metabolic, or neoplastic disorders. The present study aimed to identify the etiological spectrum of splenomegaly among children admitted to the pediatric ward of a tertiary care hospital. The present study also sought to analyze the clinical profile, associated systemic findings, grading patterns, and assess treatment response and outcomes, thereby identifying region-specific trends that may guide early diagnosis and management strategies. Despite earlier studies addressing pediatric splenomegaly in different Indian populations, data from this coastal Andhra Pradesh region remain limited. Variations in endemic infection patterns, hematologic disorders, and socioeconomic determinants necessitate region-specific analysis to strengthen clinical decision-making and improve early detection strategies.

Methods

This prospective observational study included 115 children aged 0-15 years with clinically palpable splenomegaly who were admitted to the pediatric ward over a 12-month period. A detailed history and thorough physical examination were performed for all participants, and relevant investigations were conducted to determine the underlying etiology. The degree of splenic enlargement was assessed and graded using Hackett’s classification as well as conventional clinical criteria. The etiology of splenomegaly was identified, and clinical outcomes, including response to medical treatment, need for surgical intervention, recurrence, and mortality, were documented systematically.

Results

In this study of 115 children with splenomegaly, a male preponderance was observed with 73 (63.4%) males and 42 (36.6%) females. The highest incidence occurred in children under six years of age. According to Hackett’s classification, Grade III splenomegaly was the most common, seen in 51 (44.3%) patients. The most frequent presenting symptom was fever, reported in 80 (70%) children, while the most common clinical sign was pallor, observed in 58 (50.4%) patients. Infections were the leading cause of splenomegaly, accounting for 64 (55.7%) cases, followed by hematological disorders in 44 (38%) children, with storage and miscellaneous causes being less common. Regarding outcomes, the majority of children, 98 (85.2%), responded well to medical management. Splenectomy was required in five (4.3%) cases, mainly for refractory hematological disorders. Recurrence occurred in 10 (8.7%) patients, and mortality was low at two (1.7%), highlighting an overall favorable prognosis with timely diagnosis and appropriate therapy.

Conclusion

In the present study, splenomegaly in children was most commonly caused by infectious diseases, with malaria, enteric fever, and tuberculosis being the leading contributors. A male predominance was observed, and the majority of cases presented with moderate splenomegaly (Grade III). These findings underscore the importance of early recognition, prompt diagnosis, and timely management of underlying infections to prevent complications and improve outcomes in pediatric patients presenting with splenomegaly.

## Introduction

Splenomegaly is defined as an enlargement of the spleen beyond the normal limits for age and body habitus [1]. It can be detected clinically or through imaging modalities such as ultrasonography or a CT scan. As the body’s largest lymphoid organ, the spleen has a variety of immunologic functions, including acting as a sieve for the blood, removing blood cells, microorganisms, and immune complexes [1]. The spleen plays a critical role in hematological, immunological, and reticuloendothelial functions; thus, its enlargement often reflects systemic disease processes. An enlarged spleen is a frequent and important clinical sign [2]. A palpable spleen is often the earliest clinical sign of splenic enlargement and warrants further investigation, particularly if it remains palpable beyond 2 cm below the costal margin or increases progressively in size [3]. In pediatric patients, clinical assessment must account for physiological variations with age. Mild splenic enlargement may be considered normal in neonates and infants; progressive or massive enlargement requires thorough evaluation [4]. Even though a pathological spleen only becomes palpable once it reaches at least two to three times its normal size, it may be normally palpable in healthy neonates and children [5,6].

The etiology of splenomegaly in children is diverse, encompassing infections, hematological disorders, malignancies, storage diseases, portal hypertension, and autoimmune conditions [7]. The pattern varies according to age, geographic region, and endemic diseases. In developing countries, infectious causes such as malaria, enteric fever, and tuberculosis remain predominant. Hematological disorders, including thalassemia, sickle cell anemia, and leukemia, are the second most common causes in Indian children [4]. Storage disorders and autoimmune conditions are relatively rare but remain important differentials.

Recent epidemiologic research from India and Andhra Pradesh (2018-2024) highlights important shifts in the spectrum and prevalence of pediatric splenomegaly. Population-based studies from tertiary centers report a prevalence ranging from 1.4% to 2% among pediatric admissions, with most cases occurring in children under six years and a consistent male predominance. Infections, particularly malaria and viral illnesses, remain frequent, but there is increasing documentation of hematologic disorders such as thalassemia and sickle cell anemia as significant contributors, matching trends seen in regional studies and corroborating international comparisons. Despite these data, updated, localized epidemiologic data are sparse for Andhra Pradesh over the last decade. Factors, such as changing infectious disease patterns, the prevalence of inherited hemoglobinopathies, and sociodemographic shifts, may affect disease presentation and outcomes. Hence, this study aims to fill a critical knowledge gap by profiling causes, clinical features, and outcomes of pediatric splenomegaly specific to this region.

Given this diversity, understanding the regional and age-specific patterns of splenomegaly is crucial for effective diagnosis and management.

The primary objective is to determine the clinico-etiological profile of splenomegaly in children aged one month to 15 years admitted to a tertiary care hospital in coastal Andhra Pradesh. The secondary objectives are to analyze correlations between age, sex, and clinical presentation patterns; to assess associated systemic findings and categorize splenomegaly using both Hackett’s and conventional clinical grading systems; and to evaluate treatment response, recurrence, and outcome trends, thereby identifying prognostic indicators relevant to the region.

## Materials and methods

Study design and setting

This was a hospital-based prospective observational study conducted in the Department of Pediatrics at a tertiary care teaching hospital, Great Eastern Medical School and Hospital, Srikakulam, over a period of 12 months (January 2024-December 2024).

Ethical approval 

The study was approved by the Institutional Ethics Committee Great Eastern Medical School and Hospital (162/IEC/GEMS&H/2023), and informed consent was obtained from parents or guardians, in accordance with standard ethical practices and the Declaration of Helsinki. The Declaration emphasizes prioritizing participant well-being, obtaining informed consent, ensuring scientific validity, and providing additional protections for vulnerable groups such as children.

Study population

The study included a total of 115 children, aged one month to 15 years, who were admitted to the pediatric ward with clinically detectable splenomegaly on physical examination, and whose parents or guardians provided written informed consent for participation.

Children were excluded from the study if they had abdominal distension such that the spleen was not separately palpable, if they had a past history of splenectomy, or if their parents or guardians declined to provide consent.

Sample size

A sample of 115 children was obtained during the study period using a consecutive sampling method. All eligible patients presenting to the Department of Pediatrics with splenomegaly were included until the end of the 12-month study duration. Similar sample sizes and approaches were adopted in earlier regional studies on pediatric splenomegaly.

Data collection

All children underwent detailed history-taking and thorough clinical examination. Data collected included demographic information, presenting complaints, duration of illness, associated symptoms (fever, pallor, abdominal pain, jaundice, bleeding), and relevant clinical signs (hepatomegaly, purpura, growth parameters).

Hematological and other relevant investigations, like complete blood picture, peripheral smear, reticulocyte count, CRP, ESR, and abdominal ultrasonography, were performed for each child. Assessment done by two observers. Any discrepancy in grading between the two observers was resolved by joint re-examination and consensus, and the final grading was then confirmed for every patient by abdominal ultrasonography. Additional investigations were carried out as appropriate, including chest X-ray, liver function tests, renal function tests, dengue serology, malaria parasite card test, sickling test, hemoglobin electrophoresis, high-performance liquid chromatography (HPLC), serum LDH, Mantoux test, Widal test, HIV serology, bone marrow examination, skull X-ray, blood grouping, blood culture, and CT scan of the chest and abdomen. Investigations followed recommendations outlined in earlier clinical assessment protocols for pediatric splenomegaly [4].

Grading of splenomegaly was performed through detailed clinical examination and supported by relevant radiological investigations. The degree of splenic enlargement was classified according to Hackett’s clinical grading system, as presented in Table 1 [8].

**Table 1:** The enlargement of the spleen (splenomegaly) according to Hackett’s classification The grading system classifies splenomegaly from Grade 0 to Grade 5 according to the extent to which the spleen is palpable below the left costal margin. This clinical method provides a simple and reliable assessment of spleen enlargement, correlating approximately with ultrasonographic measurements.

Grade	Clinical feature
Grade 0	Spleen not palpable even on deep inspiration.
Grade 1	Spleen palpable below costal margin, usually on deep inspiration
Grade 2	Spleen palpable, but did not extend beyond a horizontal line midway between the costal margin and the umbilicus, measured along a vertical line dropped from the left nipple.
Grade 3	Spleen palpable more than halfway to umbilicus, but not below a line horizontally running through it.
Grade 4	Palpable below umbilicus but not below a horizontal line halfway between umbilicus and pubic symphysis.
Grade 5	Extending lower than class IV

The grading of splenomegaly, based on the conventional classification described in Hutchinson’s Clinical Methods, is summarized in Table 2.

**Table 2:** Grading of splenomegaly according to conventional classification Splenomegaly is categorized as mild, moderate, or massive according to the distance the spleen extends below the left costal margin on palpation, providing a practical bedside estimation of spleen size.

Grade	Clinical feature
Mild splenomegaly	1-3 cm below left costal margin
Moderate splenomegaly	>3-7 cm below left costal margin
Massive (gross) splenomegaly	>7 cm below left costal margin

Study outcomes

The potential causes of splenomegaly in children were investigated, and the outcomes of the study group were assessed in terms of complete recovery, clinical improvement, or mortality, following methodologies used in similar pediatric studies [9-12].

Outcome assessment

Complete recovery was defined as the complete resolution of presenting symptoms and normalization of both clinical and laboratory parameters at follow-up. Clinically, this included the absence of fever, abdominal pain or distension, bleeding tendency, jaundice, and other constitutional symptoms. On physical examination, complete recovery was characterized by regression or non-palpability of the spleen on abdominal examination. Laboratory evidence of recovery included normalization of hemogram parameters such as hemoglobin, total and differential leukocyte counts, and platelet counts, along with resolution of the underlying etiology, for instance, parasite clearance in malaria, negative blood cultures in bacterial infections, or improvement in liver function tests in hepatic causes.

Clinical improvement was defined as a partial resolution of symptoms and reduction in spleen size, with or without complete normalization of laboratory findings. Clinically, this corresponded to relief in major presenting symptoms such as subsidence of fever, reduction in abdominal pain, and improvement in appetite. On examination, clinical improvement was evidenced by a decrease in spleen size by at least 2 cm below the costal margin compared to baseline. Laboratory parameters showed partial improvement in blood counts or organ function tests, though complete normalization was not always achieved. 

Follow-up

A follow-up period of three months was instituted for all study participants to monitor clinical progress and treatment response. During follow-up visits, patients were reassessed for symptom resolution, spleen size reduction by physical examination, and relevant laboratory parameters to document complete recovery or clinical improvement.

Data analysis

The collected data were compiled in Microsoft Excel 2007 (Microsoft® Corp., Redmond, WA), verified for accuracy and consistency, and then analyzed using SPSS version 16.0 (IBM Corp., Armonk, NY). Comparisons between categorical variables were made using the chi-square test or Fisher’s exact test when appropriate. A p-value < 0.05 was considered statistically significant. The data were summarized using descriptive statistics such as proportions, means, and percentages, and the results were compared with findings from previous Indian and international studies.

## Results

Demographic profile

Out of 115 children studied, a male preponderance was observed, consistent with findings from other Indian series [9-11]. The majority of cases, 51 (44%) occurred in children below six years of age, which aligns with observations by Gandhari et al. and Champatiray et al., who also reported higher prevalence in younger age groups [10,11].

The majority of children were below six years of age, and a male preponderance was observed, as shown in Table 3.

**Table 3:** Age and sex distribution of study group This table shows the distribution of 115 children presenting with splenomegaly according to age and sex. A male predominance was observed, with 73 males (63.4%) and 42 females (36.5%). The majority of cases occurred in children younger than six years of age, indicating a higher prevalence of splenomegaly in early childhood.

Age group	Male	Female	Total (n = 115)	Percentage (100%)
One month to one year	8	4	12	10.43
One to five years	24	15	39	33.92
Six to 10 years	27	8	35	30.44
11-15 years	14	15	29	25.21

Clinical presentation

Fever constituted the most common presenting symptom, occurring in 80 children (70%), followed by abdominal pain in 34 children (30%) and jaundice in nine children (8%). Similar symptom profiles have been documented in prior pediatric splenomegaly studies in both Indian and global cohorts, as shown in Table 4 [9-12].

**Table 4:** Comparison of the distribution of the frequency of symptoms Fever was the most consistent and predominant presenting symptom across all Indian studies, including the present one. This uniform pattern indicates that infectious etiologies remain the leading cause of splenomegaly among Indian children. The high frequency of fever in these studies underscores the predominance of malaria, enteric fever, and viral infections as major contributing factors in developing regions, emphasizing the strong infectious burden underlying pediatric splenomegaly in India. Abdominal distension or pain occurred in approximately 34 patients(30%), while bleeding tendencies, jaundice, and weight loss/fatigue were less frequent. These findings indicate a similar clinical presentation across different pediatric populations, with fever being the predominant symptom.

Symptom	Present study (n = 115)	Kamble et al. [9] (n = 124)	Bricks et al. [12] (n = 89)
Fever	80 (70%)	93 (75%)	39 (43.8%)
Abdominal distension/pain	34 (30%)	34 (27.4%)	13 (14.6%)
Bleeding tendencies	11 (10%)	7 (5.64%)	-
Jaundice	9 (8%)	39 (31.45%)	14 (15.7%)
Weight loss/fatigue	6 (5%)	-	21 (24%)

The most common clinical signs observed in children with splenomegaly were pallor and hepatomegaly, followed by lymphadenopathy and bleeding manifestations. Associated hepatomegaly was noted in 42 children (36.5%), reflecting the overlap of systemic infectious and hematological causes, as shown in Table 5.

**Table 5:** Comparison of clinical signs and major associated etiologies in children with splenomegaly This table compares the frequency of major clinical signs observed in the present study with findings from previous studies by Kamble et al. [9] and Bricks et al. [12]. It also outlines the predominant etiological categories associated with each sign in the present cohort. Percentages represent the proportion of children exhibiting each sign. Major etiologies have been grouped as infectious, hematological, storage, or miscellaneous disorders based on clinical and laboratory correlation. Pallor noted in 58 children (50.4%) was the most frequent finding, followed by hepatomegaly noted in 42 children (36.5%), lymphadenopathy observed in 30 children (26.1%), bleeding manifestations in 12 children (10.4%), and icterus in 10 children (18.7%). The presence of hepatomegaly showed a statistically significant association with the grade of splenomegaly (χ² = 6.72, p = 0.010), indicating that children with moderate to massive splenomegaly (Hackett’s Grades III-V) were more likely to have concurrent hepatic enlargement. This suggests shared pathological mechanisms involving the reticuloendothelial system, especially in infectious and hematological disorders.

Clinical sign	Present study (n = 115)	Kamble et al. [9] (n = 124)	Bricks et al. [12] (n = 89)	Major associated etiologies in present study
Pallor	58 (50.4%)	120 (97%)	47 (53%)	Hematological disorders
Hepatomegaly	42 (36.5%)	111 (90%)	89 (100%)	Infectious causes and storage disorders
Lymphadenopathy	30 (26.1%)	29 (23.38%)	9 (10%)	Infectious and hematological causes
Icterus	10 (8.7%)	39 (31.45%)	8 (9%)	Hepatic and hemolytic disorders
Bleeding manifestations	12 (10.4%)	7 (5.64%)	-	Hematological disorders and infections

Etiological profile

Infectious diseases were the predominant cause of splenomegaly, with malaria, enteric fever, and tuberculosis contributing the largest share, as shown in Table 6. This pattern is consistent with prior studies from endemic regions of India, which highlight infectious etiologies as the leading contributors [10,11]. Hematological disorders, including hemolytic anemia and sickle cell disease, accounted for a smaller but significant proportion, similar to reports by Rajanna et al. [2]. Storage disorders and other rare causes were identified in a minority of cases, in line with international data.

**Table 6:** Etiological profile of splenomegaly This study included 115 children with splenomegaly, categorized according to major etiological groups. Infections were the most common cause, observed in 64 (55.7%) patients, followed by hematological disorders in 44 (38.3%). Storage disorders and miscellaneous causes were relatively uncommon, accounting for two (1.7%) and five (4.3%) cases, respectively. This table shows the distribution of 115 children with splenomegaly according to the major etiological categories. Among infectious causes, malaria was seen in 23 (20%) patients, enteric fever in 18 (15.7%), viral infections in 17 (14.7%), and tuberculosis in 6 (5%). Among hematological disorders, hemolytic anemia accounted for 32 (28%) cases and leukemia for 12 (10.3%). A significant association was found between infectious etiologies and mild-to-moderate splenomegaly, whereas hematological causes were more frequently linked to massive splenomegaly (Grades IV-V) (χ² = 8.52, p = 0.004), suggesting differing disease mechanisms and chronicity patterns.

Etiological category	Diseases	Number of cases (n = 115)	Percentage (100%)
Infections	-	Total = 64	Total = 55.7
	Malaria	23	20
	Enteric	18	15.7
	Viral	17	14.8
	Tuberculosis	6	5.2
Hematological	-	Total = 44	Total = 38.3
	Hemolytic anemia	32	28
	Leukemia	12	10.3
Storage disorders		2	1.7
Miscellaneous		5	4.3

**Figure 1:**
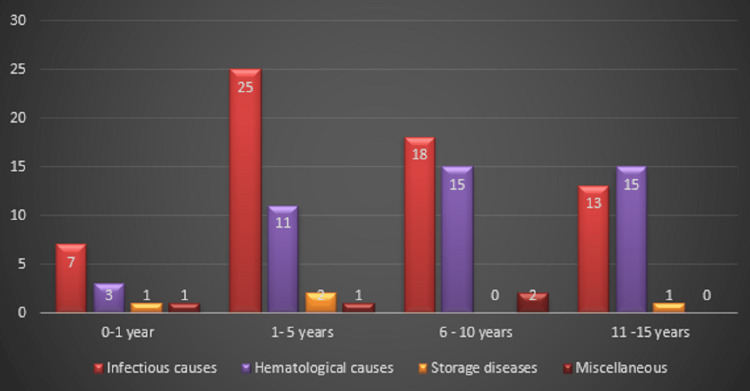
Age-wise distribution of etiological groups The bar chart illustrates the relationship between age groups and underlying etiological categories. Infectious causes were predominant in younger children, particularly in the one to five years age group, while hematological causes were more frequent among older children (six to 15 years). Storage and miscellaneous causes were comparatively rare and evenly distributed across age groups.

In the present study, the majority of cases (33.9%) were observed in the one to five years age group, followed by six to 10 years (30.4%) and 11-15 years (25.2%), while infants (<1 year) accounted for 10.4% of the total cases. Infectious causes predominated across all age groups, being most frequent in children aged one to five years (39.7%) and six to 10 years (28.6%). Hematological etiologies were more common among older children, particularly in the six to 10 years (34.1%) and 11-15 years (34.1%) groups. Storage and miscellaneous causes were relatively rare and distributed sporadically across age categories, with a slight clustering in the younger age group (<5 years), as shown in Figure 1.

Infectious and hematological causes exhibited an inverse relationship with age: infectious etiologies were more common in younger children, whereas the prevalence of hematological causes increased with advancing age, as illustrated in Table 7.

**Table 7:** Age-wise distribution within etiological groups This study of 115 children with splenomegaly analyzed the distribution of underlying etiologies across age groups. Infectious diseases were the most common cause, seen in 64 (54.8%) children, particularly in the one- to five-year age group, where 25 (39.7%) cases occurred. Hematological disorders were observed in 44 (38.3%) patients and were more frequent among children aged six to 15 years. Storage and miscellaneous disorders were relatively uncommon and did not show a consistent age-related pattern. Statistical analysis using the chi-square test indicated no significant association between age group and etiology (p = 0.287).

Age group	Infectious (n = 63)	Hematological (n = 44)	Storage (n = 4)	Miscellaneous (n = 4)	Total (n = 115)
<1 year (12)	7 (11.1%)	3 (6.8%)	1 (25.0%)	1 (25.0%)	12 (10.4%)
One to five years (39)	25 (39.7%)	11 (25.0%)	2 (50.0%)	1 (25.0%)	39 (33.9%)
Six to 10 years (35)	18 (28.6%)	15 (34.1%)	0 (0%)	2 (50.0%)	35 (30.4%)
11-15 years (29)	13 (20.6%)	15 (34.1%)	1 (25.0%)	0 (0%)	29 (25.2%)

Table 8 presents a comparative analysis of the etiological profiles of splenomegaly as reported in this study, alongside findings from other published studies.

**Table 8:** Comparison of etiological profile with other studies This table compares the etiological spectrum of pediatric splenomegaly in the present study with findings from previous Indian and global studies. Infectious causes - predominantly malaria, enteric fever, and tuberculosis - remained the leading etiologies, followed by hematological disorders such as hemolytic anemia and leukemia. The association between hematological etiologies and higher grades of splenomegaly was statistically significant (χ² = 8.52, p = 0.004), indicating that chronic hematological disorders contribute to more pronounced splenic enlargement.

Etiology	Present study (n = 115)	Kamble et al. [9] (n = 124)	Champatiray et al. [11] (n = 150)	Bricks et al. [12] (n = 89)
Infections	64 (55.7%)	20 (16.1%)	75 (50%)	49 (54.8)
Hematological	44 (38.3%)	100 (80.64%)	54 (36%)	32 (36.5)
Storage disorders	2 (1.7%)	1 (0.8%)	2 (1.3%)	3 (3.8)
Miscellaneous	5 (4.3%)	3 (2.4%)	19 (12%)	5 (4.9)

Distribution of patients according to the grading of splenomegaly

According to Hackett’s classification, Grade III splenomegaly was the most frequent clinical grade, observed in 51 (44.3%) patients, followed by Grade II in 23 (20%) and Grade I in 18 (15.7%) children. This distribution is similar to that reported in malaria-endemic regions, where moderate splenomegaly predominates, as shown in Table 9 [9,11].

**Table 9:** Distribution of patients according to Hackett’s classification The distribution of splenomegaly grades among 115 children showed that Grade III was the most common, observed in 51 (44.3%) patients, followed by Grade II in 23 (20%) and Grade I in 18 (15.7%). Higher grades were less frequent, with Grade IV seen in 14 (12.2%) and Grade V in nine (7.8%) cases, reflecting the predominance of mild-to-moderate splenic enlargement in this pediatric cohort.

Grade of splenomegaly	Number of patients (n = 115)	Percentage (100%)
Grade I	18	15.7
Grade II	23	20
Grade III	51	44.3
Grade IV	14	12.2
Grade V	9	7.8

Table 10 provides a comparison of splenomegaly grading observed in this study with the grading systems and findings reported in other studies.

**Table 10:** Comparison of splenomegaly (as per conventional classification) with other studies In the present study, comprising 115 pediatric cases of splenomegaly, moderate (Grade III) enlargement of the spleen was the most frequently observed, seen in 51 (44%) of cases. Mild splenomegaly (Grades I-II) was noted in 41 (36%), while massive splenomegaly (Grades IV-V) accounted for 23 (20%) of the cases. The present study shows a distribution pattern of splenomegaly grades that is largely consistent with previous reports by Kamble et al. [9] and Gandhari et al. [10]. Mild splenomegaly (Grades I-II) accounted for a smaller yet significant proportion of cases across all studies, while massive splenomegaly (Grades IV-V) constituted the least common presentation. This consistent trend suggests that moderate enlargement of the spleen represents the typical clinical presentation pattern in pediatric and general populations studied, irrespective of regional variations.

Grade	Present study (n = 115)	Kamble et al. [9] (n = 124)	Gandhari et al. [10] (n = 100)
Grades I-II (mild)	41 (36%)	46 (37.09%)	15 (15%)
Grade III (moderate)	51 (44%)	41 (33.06%)	62 (62%)
Grades IV-V (massive)	23 (20%)	37 (29.83%)	23 (23%)

Outcome and response to treatment

With appropriate management, 98 (85.2%) of children achieved complete recovery or significant improvement. These outcome rates are comparable to those reported in other Indian studies, where mortality ranged from 2-5% [9,11]. The recurrence rate was low, as shown in Table 11.

**Table 11:** Outcome/response to treatment This study of 115 children with splenomegaly evaluated treatment outcomes stratified by spleen size: mild in 41 (36%), moderate in 51 (44%), and massive in 23 (20%). The majority of patients responded favorably to conservative management, with 98 (85.2%) achieving complete resolution of symptoms with medical treatment (p = 0.021). Splenectomy was required in only five (4.3%) cases, predominantly among those with moderate (n = 3) and massive (n = 2) splenomegaly, showing a significant association (p = 0.032). Recurrence or relapse occurred in 10 (8.7%) patients, with no significant correlation to spleen size (p = 0.437). Mortality was low, reported in two (1.7%) children with massive splenomegaly, which was not statistically significant (p = 0.587). These findings indicate that most children with splenomegaly respond well to medical therapy, and timely diagnosis with appropriate etiological management can lead to favorable outcomes while minimizing the need for surgical intervention.

Outcome/response	Mild (n = 41)	Moderate (n = 51)	Massive (n = 23)	Total (n = 115)	Percentage (100%)	p-value
Resolved with medical treatment	36	43	19	98	85.3%	0.021
Required surgery (splenectomy)	0	3	2	5	4.3%	0.032
Recurrence/relapse	5	5	0	10	8.7%	0.437
Mortality	0	0	2	2	1.7%	0.587

All patients completed the study follow-up without any dropouts, ensuring complete data capture for outcome analysis. 

## Discussion

Splenomegaly in children is a clinical manifestation of diverse systemic illnesses, predominantly of infectious and hematological origin in developing countries [10,11]. The present study was primarily designed to construct an etiological profile of pediatric splenomegaly in this region, with secondary aims focused on analyzing age- and sex-related associations, clinical grading correlations, and treatment outcomes.

Historically, congestive and hematological causes were considered the most frequent, but recent studies highlight infections as the leading etiology in endemic regions [10,11].

In this prospective study of 115 pediatric patients with splenomegaly, 51 (44.3%) were below six years of age, including 12 (10.4%) infants (<1 year) and 39 (33.9%) children aged one to five years, highlighting the substantial burden of splenomegaly in early childhood. Comparable findings were reported by Gandhari et al., with 48% of cases under six years, and Champatiray et al., with 45% in the same age group [10,11].

A male predominance was noted, with 73 (63.5%) males and 42 (36.5%) females (M:F ratio 1.7:1), consistent with other Indian studies [9-11]. This pattern may reflect greater exposure of males to infectious agents or sociocultural factors influencing healthcare access.

The most common presenting symptom in our cohort was fever, followed by abdominal pain, while pallor and hepatomegaly were the most frequent signs. This aligns with prior observations in similar pediatric populations [9-12].

Ultrasonography was performed in all cases in addition to clinical evaluation to confirm the spleen grade and to assist in etiological evaluation, aligning with current recommendations for the assessment of pediatric splenomegaly. Ultrasonography is an established, safe, fast, and reliable method for the measurement of spleen size [13]. Moderate (Grade III) splenomegaly was the most common presentation, followed by mild and massive grades.

Infectious etiologies were the predominant cause of splenomegaly, accounting for 64 (55.7%) cases. Among these, malaria was observed in 23 (20%), enteric fever in 18 (15.7%), and tuberculosis in six (5%) patients. These findings are comparable to those reported by Gandhari et al., where infections constituted 50% of cases, with malaria accounting for 25%, and by Champatiray et al., who reported 52% infectious causes, including malaria in approximately 29% of cases [10,11]. Similarly, Bricks et al. (47.2%) also demonstrated an infectious predominance [12]. In contrast, Kamble et al. reported hematological disorders - primarily hemolytic anemia and thalassemia - as the leading etiology, accounting for 80.6% of cases, highlighting regional variations in hemoglobinopathy prevalence [9].

International studies mirror these patterns: Suttorp et al. described hyper-reactive malarial splenomegaly in an adolescent, emphasizing immunological mechanisms, and Elmakki et al. highlighted tropical splenomegaly syndrome in endemic zones [4,14].In contrast, O’Reilly, in a series of 2,505 patients from the United States, reported hematologic, hepatic, and malignant causes as predominant, with infections forming a minority [15].

The significant association of hematological etiologies with higher grades of splenomegaly (χ² = 8.52, p = 0.004) aligns with the chronic nature of these disorders, causing sustained splenic enlargement. The pattern of infectious etiologies dominating younger ages and hematological conditions increasing with age supports age-specific diagnostic considerations, reinforcing the importance of thorough hematological workups in older children presenting with massive splenomegaly.

Most children, 98 (85.2%), responded favorably to medical management, underscoring the effectiveness of timely diagnosis and appropriate treatment, particularly for infectious causes. Splenectomy was required in 10 (8.7%) cases, primarily for refractory hematological disorders. Recurrence occurred in five (4.3%) patients, reflecting persistence or reactivation of underlying conditions such as malaria or hemoglobinopathies. Mortality was low, reported in two (1.7%) cases, likely attributable to improved diagnostic facilities, enhanced antimicrobial therapy, and better supportive care. These findings emphasize that pediatric splenomegaly generally carries a favorable prognosis when managed promptly and appropriately [9,12].

In summary, this study demonstrates that infectious diseases remain the predominant cause of pediatric splenomegaly in developing countries, particularly in children under six years.

Hematological causes of splenomegaly are relatively common in this region due to several interrelated factors. The high prevalence of hemoglobinopathies, including β-thalassemia, sickle cell disease, and other inherited disorders, particularly in Eastern India and Andhra Pradesh, leads to chronic hemolysis and subsequent splenic enlargement. Nutritional deficiencies, such as iron, folate, and vitamin B12 deficiencies, are also frequent and contribute to chronic anemia, stimulating extramedullary hematopoiesis and further splenomegaly. Endemic infections, including malaria, kala-azar, and enteric fever, may unmask or exacerbate underlying hematological conditions. In some communities, consanguineous marriages increase the likelihood of homozygous hemoglobinopathies, which often present with early-onset splenomegaly. Additionally, limited access to early diagnosis and screening delays recognition of these conditions, resulting in children presenting with moderate to massive splenomegaly. Together, these factors explain the relatively high frequency of hematological disorders as a secondary cause of pediatric splenomegaly in this population.

Hematological and storage disorders represent secondary contributors. Early recognition, appropriate investigations, and timely treatment are crucial to reducing morbidity and mortality.

Limitations

Limitations of the study include the relatively small sample size from a single tertiary care center, which may limit the generalizability of the findings to broader populations. Some diagnostic tests were performed based on clinical judgment, potentially introducing variability in etiological classification. The three-month follow-up, while adequate for assessing short-term outcomes, may not capture long-term sequelae or relapses.

## Conclusions

This study shows that infectious diseases remain the leading cause of pediatric splenomegaly in developing regions, particularly in younger children, while hematological disorders are more common in older age groups and often associated with severe presentations. The findings reflect regional epidemiological patterns, with infections predominating in low-resource settings and non-infectious causes more frequent in higher-income areas. Accurate clinical grading and ultrasound assessment are essential for early diagnosis and appropriate management. Larger, multicentric studies are needed to validate these observations and refine context-specific strategies for improving outcomes.
